# Risk factors for death in patients with non-infectious adverse events

**DOI:** 10.1590/1518-8345.2069.3001

**Published:** 2018-07-16

**Authors:** Gilcilene Oliveira Gadelha, Hémilly Caroline da Silva Paixão, Patricia Rezende do Prado, Renata Andréa Pietro Pereira Viana, Thatiana Lameira Maciel Amaral

**Affiliations:** 1 RN, Unidade Básica de Saúde Francisco Taveira, Secretaria Municipal de Saúde, Senador Guiomard, AC, Brazil.; 2 RN.; 3 PhD, Adjunct Professor, Universidade Federal do Acre, Rio Branco, AC, Brazil.; 4 PhD, RN, Hospital do Servidor Público, São Paulo, SP, Brazil.

**Keywords:** Critical Care, Intensive Care Units, Risk Factors, Mortality, Patient Safety, Nursing

## Abstract

**Objetive:**

to identify risk factors for death in patients who have suffered non-infectious adverse events.

**Method:**

a retrospective cohort study with patients who had non-infectious Adverse Events (AE) in an Intensive Care Unit. The Kaplan Meier method was used to estimate the conditional probability of death (log-rank test 95%) and the risk factors associated with death through the Cox regression.

**Results:**

patients over 50 years old presented a risk 1.57 times higher for death; individuals affected by infection/sepsis presented almost 3 times the risk. Patients with a Simplified Acute Physiology Score III (SAPS3) greater than 60 points had four times higher risk for death, while those with a Charlson scale greater than 1 point had approximately two times higher risk. The variable number of adverse events was shown as a protection factor reducing the risk of death by up to 78%.

**Conclusion:**

patients who had suffered an adverse event and who were more than 50 years of age, with infection/sepsis, greater severity, i.e., SAPS 3>30 and Charlson>1, presented higher risk of death. However, the greater number of AEs did not contributed to the increased risk of death.

## Introduction

Adverse Event (AE) is understood as the unintentional injury caused to the patient, not related to the underlying morbidity, as a result of the interventions of the health team and that can generate prolongation of the hospitalization time, suffering, physical and emotional discomfort, incapacity and death^1^.

Patients hospitalized in Intensive Care Units (ICUs) are particularly vulnerable and susceptible to the occurrence of these injuries due to the severity of their clinical condition, the instability of their condition, the need for constant and numerous emergency interventions performed by the multidisciplinary team involved in the assistance, as well as the large number of diagnostic procedures and the use of specific and complex drugs^2^ ^-^ ^5^.

Studies on adverse events in patients admitted to ICUs have become more prominent in publications since 1995. It is worth noting the study performed in an ICU of a hospital in Jerusalem, which verified the occurrence of 1.7 errors for each patient per day, which occurred in an average of 178 activities performed by practitioners involved in the care, and 29% of these errors were classified as probable cause of serious clinical complications or even death^6^. 

In a study carried out in Belgium in 2012, it was verified that the percentage of death ranged from 0% to 58% and the length of stay in the ICU ranged from 1.5 to 10.4 days^7^. In the US, in 2013, between 210,000 and 440,000 deaths were associated with adverse events and almost half of these could have been avoided^8^, and in 2016 the Leapfrog Group estimated 206,201 avoidable deaths, with 33,439 lives that could be saved each year if all hospitals had a good performance regarding the safety of their patients^9^. Also in 2016, Makary and Daniel’s report to Johns Hopkins University estimated that the number of avoidable deaths was estimated at more than 250,000^10^. 

In Latin America, according to data collected from 58 hospitals, the prevalence of adverse events was 10.5% (95% CI 9.91 to 11.04), with 28% resulting in disability and 6% in the death of the patient. It is worth mentioning that 60% of AE were considered avoidable^11^. In Brazil, in 2009, the incidence of AE found was 7.6%, and in 2011, 2.9% of events were associated with death of patients^12^ ^-^ ^13^. 

In Rio de Janeiro, the main types of non-infectious adverse events were due to delayed or failed diagnosis and/or treatment and development of pressure ulcers^14^. At the same city, a prospective cohort study in an intensive care unit found that the incidence rate of adverse events was 9.3 per 100 patient/day and the occurrence of adverse event resulted in a 19-day increase in length of stay and doubled the chance of the individual evolving to death (OR=2.047, 95% CI: 1.172-3.570)^15^. 

Non-infectious adverse events represent 72.4% of all events, most of which are associated with invasive procedures^16^. In Brazil, there are few studies on the subject, so it is necessary to conduct further investigations in the different regions of the country. In this context, the present study aims to identify risk factors for death in patients who have suffered non-infectious adverse events in an Intensive Care Unit. 

## Method

A retrospective cohort study of patients who had suffered non-infectious adverse events (AE) during hospitalization in an Intensive Care Unit (ICU) of Rio Branco, Acre, in the period from September 2012 to July 2014.

The study sample consisted of the total number of patients admitted to the ICU, aged 18 years or more, and the follow-up period was the period from admission to hospital discharge, referral or death. 

Non-infectious adverse events were considered: a) medication: missed dose, wrong dose, wrong concentration, wrong medication, wrong route of administration, wrong speed, wrong time, wrong patient; b) endotracheal tube (oro/nasotracheal) or tracheostomy (obstruction, unscheduled withdrawal, disconnection, incorrect position, incorrect fixation and others); c) probes, drains and catheters: (oro/nasogastric) probe, gastrostomy or jejunostomy, permanent vesical catheter, ureterostomy or cystostomy, drainage and central, peripheral, arterial and pulmonary catheters (obstruction, unscheduled withdrawal, inadequate position, inadequate output measurement, inadequate fixation and others); d) pressure ulcer (PU), with damage and prolongation of hospitalization and without damage, but with some intervention; e) fall: of the bed, stretcher, chair or of one’s own height.

The concept of non-infectious adverse event, used in the present research, was “the set of errors actually occurred (generating or not non-infectious damage to the patient) and all non-infectious damage related to the care process”^17^.

In the analysis of the adverse events occurred due to medication the main drugs and the severity of the event were detailed; and in the event of pressure ulcer, the main place of occurrence was highlighted. The number of events occurred in the same patient was also evaluated, as well as the types of events.

The data were collected from the electronic medical records database of the adult ICU, being evaluated the occurrence or non-occurrence of adverse events. The independent variables included were sex, diagnosis of ICU admission, age, type of hospitalization (clinical or surgical), length of stay, systolic and diastolic blood pressure in the first hour, serum lactate and creatinine levels, presence of mechanical ventilation, use of vasopressors, Glasgow coma scale, SAPS3 prognosis and Charlson score^12^. 

To evaluate the Simplified Acute Physiology Score III (SAPS3), a severity score, we analyzed the demographic variables, the comorbidities, some specific diagnoses, the use of invasive support, as well as physiological and laboratorial variables present in ICU admission^18^. Through SAPS3, a score is obtained from which the probability of hospital death is estimated. In the interpretation of the score, we consider that the highest number of points, the higher the severity of the patient^12^. Also to express the severity profile due to comorbidities, the Charlson score was used, in which scores were assigned from one to six for the 17 clinical conditions^12^. 

For the analysis of the defined variables, the descriptive statistics and the association measures were used. In the description of the continuous variables, the measures of central tendency (mean and standard deviation) were presented and the categorical variables were expressed by absolute and relative frequency distribution. In the comparison of groups with and without the occurrence of non-infectious adverse events, unpaired Student’s t-tests and Pearson’s chi-square test were performed, considering the nature of the continuous and categorical variables, respectively. 

In order to evaluate the risk of death among the patients who had suffered AE, the zero time (T0) of the cohort was defined as the date of the occurrence of the AE, and the follow-up time (ΔT) was the time elapsed between T0 and the outcome (discharge or death). The Kaplan Meier method was used to estimate the conditional probability of death on the 12th and 24th day of follow-up, using the 95% log-rank test to evaluate the differences between the curves.

Gross and adjusted Cox regression models, with their respective 95% confidence intervals, estimated the risk factors for death. The final model was built to evaluate the prognostic factors for death in patients who had suffered non-infectious AE in the ICU. The independent variables that demonstrated statistical significance by the univariate analysis were included in the multivariate Cox regression model, with p-value <5% of input and p-value> 10% as exclusion criterion for the model. 

The data were organized in an Excel spreadsheet of the Microsoft Office^®^ 2010 package (Microsoft, USA) and analyzed with SPSS^®^, version 17.0 (SPSS Corp, Chicago, USA). In all analyzes, we adopted the level of significance of a = 5%.

This study was approved by the Research Ethics Committee of the Federal University of Acre under opinion No. 1,336,173.

## Results

Among the 792 patients admitted to the ICU in the period evaluated, 36.2% had some type of non-infectious AE, the majority being male and older than 50 years. The variables length of stay in the ICU, type and reason for hospitalization, use of mechanical ventilation and vasopressor drugs, score of less than eight points in the Glasgow coma scale and higher mortality risk measured by SAPS3 showed statistically significant differences between individuals who had suffered non-infectious AEs when compared to those who had not (Table 1). 

**Table 1:** Clinical and epidemiological characteristics of patients with and without the occurrence of non-infectious adverse events in a Intensive Care Unit of Rio Branco, Acre, Brazil, 2012-2014

Variable		Total	Without AE*	With AE*	p-value^†^
		n(%)	n(%)	n(%)	
Age					0.063
	<50	410 (51.8)	274 (54.3)	136 (47.4)	
	50 or more	382 (48.2)	231 (45.7)	151 (52.6)	
Sex					0.284
	Male	466 (58.8)	290 (57.4)	176 (61.3)	
	Female	326 (41.2)	215 (42.6)	111 (38.7)	
Length of stay in the ICU^‡^					<0.001
	≤ 7 days	458 (58.3)	390 (77.4)	68 (24.1)	
	8-15 days	183 (23.4)	90 (17.8)	93 (33.0)	
	>15 days	145 (18.3)	24 (4.8)	121 (42.9)	
Hospitalization					0.028
	Clinical	572 (72.2)	378 (74.9)	194 (67.6)	
	Surgical	220 (27.8)	127 (25.1)	93 (32.4)	
Reason for hospitalization					<0.001
	Cardiovascular disorders	123 (15.5)	101 (20.0)	22 (7.7)	
	Infection/sepsis	101 (12.8)	63 (12.5)	38 (13.2)	
	Neurological disorders	145 (18.3)	95 (18.8)	50 (17.4)	
	Other clinical changes	135 (17.0)	84 (16.6)	51 (17.8)	
	Surgery	167 (21.1)	101 (20.0)	66 (23.0)	
	Trauma	121 (15.3)	61 (12.1)	60 (20.9)	
Mechanical ventilation					<0.001
	No	399 (50.4)	289 (57.2)	110 (38.3)	
	Yes	393 (49.6)	216 (42.8)	177 (61.7)	
Vasopressors					<0.001
	No	501 (63.3)	348 (68.9)	153 (53.3)	
	Yes	291 (36.7)	157 (42.8)	134 (46.7)	
Glasgow Coma Scale					<0.001
	<8 points	396 (52.4)	218 (45.4)	178 (64.5)	
	≥8 points	360 (47.6)	262 (54.6)	98 (35.5)	
SAPS3^§^					0.024
	≤30	485 (61.2)	331 (65.5)	154 (53.7)	
	31-60	196 (24.7)	105 (20.8)	91 (31.7)	
	>60	111 (14.1)	69 (13.7)	42 (14.6)	
Outcome					0.171
	Discharge	553 (70.4)	363 (72.0)	190 (67.4)	
	Death	233 (29.6)	141 (28.0)	92 (32.6)	
Charlson (points)					0.440
	0	552 (71.1)	351 (70.2)	201 (72.8)	
	1 and more	224 (28.9)	149 (29.8)	75 (27.2)	
		Mean±SD	Mean±SD	Mean±SD	p-value^†^
	Systolic BP^║^ in the 1st hour	105.1±21.8	105.3±23.2	104.7±19.2	0.076
	Diastolic BP^║^ in the 1st hour	65.8±16.4	66.3±17.5	65.1±14.1	0.304
	Serum lactate	9.2±9.9	9.3±10.0	8.9±9.9	0.105
	Serum creatinine in the 1st hour	1.7±3.4	1.9±3.9	1.3±2.3	0.074
	Total	792 (100.0)	505 (63.8)	287 (36.2)	

*AE: Adverse event; †p-value: Chi-square test; ‡ICU: Intensive Care Unit; §SAPS3: Simplified Acute Physiology Score;║BP: Blood Pressure

Of the total of patients who had suffered non-infectious AEs, 43.6% had suffered more than one event. Among the AEs, the pressure ulcers represented almost half of the events, being located mainly in the sacral and calcaneal regions, followed by the use of probes, drains and catheters, and those related to medication and blood transfusion (Table 2). 

Still on drug-related AEs, the majority occurred in the administration of antimicrobials, with the most frequent errors being the non-administration and the application of the wrong drug, approximately 60% considered serious or very serious (Table 2).

Individuals aged 50 years or older had a higher conditional probability of death, reaching almost 42.0% within 24 days of hospitalization. The use of probes, drains and catheters, pressure ulcers and the occurrence of three or more non-infectious AEs per patient resulted in a greater probability of death at 12 and 24 days (Table 3).

**Table 2:** Description of non-infectious adverse events suffered by patients from an Intensive Care Unit of Rio Branco, Acre, Brazil (2012-2014)

Variable		N*	%
AE^†^ per patients (n=287)			
	01 AE^†^	162	56.4
	02 AE^†^	62	21.6
	03 or more AE^†^	63	22.0
Type of AE^†^ (n=532)			
	Pressure ulcers	227	42.7
	Probes, drains and catheters	142	26.7
	Medication and blood transfusion	94	17.7
	Endotracheal tube, tracheostomy, barotrauma and reintubation	61	11.5
	Fall	08	1.4
Localization of the pressure ulcers (n=227)			
	Sacrum	117	51.5
	Calcaneus	58	25.5
	Scalp	26	11.5
	Others	26	11.5
Drugs related to AE^†^ (n=91)			
	Antimicrobials	40	43.9
	Anti or procoagulants	16	17.6
	Sedation/analgesia	07	7.7
	Electrolytes	05	5.5
	Insulin	03	3.3
	Vasopressors/catecholamines	03	3.3
	Others	17	18.7
Reason for drug-related AEs^†^ (n=91)			
	Non-administration	46	50.5
	Wrong drug	18	19.8
	Wrong dose	08	8.8
	Wrong time	07	7.7
	Wrong prescription	06	6.6
	Wrong patient	03	3.3
	Others	03	3.3
Severity of drug-related AEs^†^ (n=91)			
	Light	19	20.9
	Mild	19	20.9
	Serious	47	51.6
	Very serious	06	6.6

*N: missings; †AE: Adverse event

**Table 3:** Survival according to the clinical and epidemiological characteristics of patients who had suffered non-infectious adverse events in an Intensive Care Unit of Rio Branco, Acre, Brasil, 2012-2014

Variable		SVV(%)*		Log-Rank
		12 d	24 d	p-value
Age				0.032
	<50 years old	16.9	28.7	
	50 or more	19.3	41.8	
Sex				
	Male	16.3	39.0	0.224
	Female	21.5	45.1	
Hospitalization				0.052
	Clinical	19.1	43.0	
	Surgical	18.0	26.6	
Reason for hospitalization				0.063
	Trauma	9.7	17.5	
	Cardiovascular disorders	12.3	62.1	
	Infection/sepsis	32.5	50.0	
	Neurological disorders	20.5	43.8	
	Other clinical changes	15.7	43.1	
	Surgery	21.5	31.4	
Type of AE^†‡^				
	Medication and blood transfusion	18.7	39.5	0.993
	Endotracheal tube, tracheostomy, barotrauma and reintubation	14.7	45.3	0.420
	Probes, drains and catheters	10.8	26.0	0.025
	Pressure ulcers	14.2	29.3	0.001
	Falls	-	-	-
Number of AE per patients				<0.001
	01 AE^†^	28.2	50.7	
	02 AE^†^	11.1	30.3	
	03 or more AE^†^	3.6	22.6	

* SVV: Survival, Kaplan Meier Method; †AE: Adverse event; ‡It does not add 100.0% since the same individual can suffer more than one type of adverse event

Individuals aged 50 years of age or older who has suffered AEs had an increased risk of death by 57.0% when compared to younger patients, whereas the risk of death was almost three times greater among patients hospitalized due to infection or sepsis compared to those hospitalized due to trauma. Patients with AE who had scores above 60 points on SAPS3 had a four times higher risk of death compared to patients with scores of 30 points below, whereas those with 1 point or higher on the Charlson scale had two times greater risk for death than those who had not scored. Interestingly, patients who had 3 or more non-infectious adverse events had the risk of death reduced by 78.0% compared with those who had only one AE (Table 4).

**Table 4:** Gross and adjusted Hazard Ratio (HR) of the prognostic factors of death among patients who had suffered non-infectious adverse events in an intensive care unit of Rio Branco, Acre, Brasil, 2012-2014

Variable		Hazard Ratio gross (95% CI)	Hazard Ratio adjusted (95% CI)*
Age			
	<50 years old	1.00	1.00
	50 or more	1.62 (1.05-2.49)	1.57(1.01-2.43)
Sex			
	Male	1.00	1.00
	Female	0.77 (0.51-1.17)	0.84 (0.55-1.29)
Hospitalization			
	Clinical	1.00	1.00
	Surgical	1.57 (0.99-2.48)	1.42 (0.89-2.27)
Reason for hospitalization			
	Trauma	1.00	1.00
	Cardiovascular disorders	2.20 (0.85-5.70)	1.76 (0.65-4.77)
	Infection/sepsis	3.07 (1.43-6.59)	2.62 (1.18-5.78)
	Neurological disorders	2.03 (0.97-4.27)	1.72 (0.79-3.75)
	Other clinical changes	1.73 (0.80-3.75)	1.41 (0.62-3.20)
	Surgery	1.45 (0.70-3.02)	1.32 (0.63-2.78)
AE^†^ per patients^‡^			
	01 AE^†^	1.00	1.00
	02 AE^†^	0.45 (0.25-0.77)	0.52 (0.29-0.93)
	03 or more AE^†^	0.23 (0.13-0.43)	0.22 (0.11-0.41)
SAPS3^§^			
	≤30	1.00	1.00
	31-60	2.19 (1.34-3.60)	2.12 (1.27-3.51)
	>60	4.40 (2.57-7.51)	4.18 (2.37-7.37)
Charlson (points)			
	0	1.00	1.00
	1 and more	2.27 (1.49-3.48)	2.12 (1.31-3.43)

*Adjustment variables: age and sex; †AE: Adverse event; ‡Adjustment variables: age, sex, type of hospitalization, reason for hospitalization; §SAPS3: Simplified Acute Physiology Score

## Discussion

The present study points out that the risk of death in patients with non-infectious AEs is not due to the number of events that the patient suffered, but rather to the severity of the patient and to the presence of comorbidities. It was also observed that, when comparing individuals with and without non-infectious AEs, the length of stay, the type and reason for hospitalization, and the variables related to patient severity (SAPS3, Glasgow, use of mechanical ventilation and vasopressor) presented statistically significant differences. This result is consistent with both the international and national literature and the explanation is linked to a prolonged stay, presence of comorbidities and greater severity of the patients^3^ ^,^ ^5^.

This study identified 36.2% of cases of AE in the analyzed patients, a figure higher than that found in countries such as Canada, the United States and France, where 19%, 20.2% and 31% of patients had suffered at least one AE, respectively^19^ ^-^ ^20^. We must take into account that the number of nursing professionals per bed in ICUs in Europe ranges from two to three patients for each nurse, whereas in the Brazilian intensive care units it corresponds to ten patients for each nursing professional. We can propose, in view of the results quoted, that this number is not recommendable and interferes in the quality of care^21^ ^-^ ^22^. A recent resolution of the Federal Nursing Council, no. 543/2017, indicates 1.33 patients per nursing professional, close to the international reality, being a challenge to be reached in the intensive care units in the North of the country^23^. 

In France, in 2010, a study that analyzed the incidence of medical errors and their relationship with mortality in 70 ICUs found that the error rate was equivalent to 2.1/1000 patients each day, of which 15.4% represented adverse events with a considerable increase in mortality for those individuals who had suffered more than two AEs^24^.

It is worth mentioning that in the studied ICU the increase in the number of AEs represented a protection factor probably related to the greater non-infectious post-AE care adopted by the team, as well as the greater attention given to these patients because they are patients with greater severity. 

In 2014, in Japan, a study to verify the influence of AEs occurred due to use of drugs found that 15% were drug-related, with an incidence of 30.6 per 1000 patients per day, of which 70% were 65 years or more and the mean length of saty of individuals who had suffered at least one drug-related AE was 13 days^25^.

In Brazil, a study carried out in the state of São Paulo, in 2014, demonstrated that the most common AEs, which are under the responsibility of the nursing staff, were dermatitis, rash and pressure ulcer, corresponding to 60.4%, thus evidencing, as in our study, that skin injuries were the most found AEs^5^.

In Piauí, 29% of the patients presented pressure ulcers, 58.3% of which were located in the sacral region^26^. These high figures, especially with regard to the location in the sacral region, suggest that measures for pressure ulcer prevention have not been adequately observed by the health team.

Thus, it is necessary to adopt measures to prevent pressure ulcers. In addition to providing an adequate nutritional contribution, body hygiene and use of moisturizers and humectants for skin, it is of great importance to protect bony protrusions, identify risk factors targeting treatment, make record of skin changes, make use of risk assessment scale, change of decubitus every two hours and, among others, monitor and register interventions and their respective results^27^.

In Canadian and North American studies^19^ ^-^ ^20^, the drug-related AEs were the second largest group, with 21% and 20.2%, respectively. In the city of Ribeirão Preto, SP, the incidence of drug-related adverse events in the ICU was 18.9%, of which the wrong time corresponded to 35.3%, wrong annotation to 23.5%, wrong dose to 17.6%, and dose omission to 5.9%^28^. However, in the present study, dose omission corresponded to 50.5% of drug events. Due to the wide variety of drug-related AEs, there is a need to monitor these errors, as well as to identify the factors related to their occurrence. In addition, the health team must observe the nine criteria for drug administration (right patient, right drug, right route, right dose, right time, right documentation, right action, right form, right response)^29^.

In 2015, a study in Jerusalem found that patients with pressure ulcer in the sacral region had lower survival rates than those without pressure ulcer (70 days versus 401 days)^30^.

A study conducted in Canada in 2008 showed that the risk of death was 1.4 times higher; in France, the risk was about twice as high in patients who presented high SAPS3, a figure below that found in our study^21^. In the present study, advanced age and the presence of infection also increased the risk that the individual affected by an AE evolved to death by 1.57 and 2.62 times, respectively. In the US, sepsis had a significant impact on the mortality of individuals over the age of 60, increasing by almost two times the risk of death, as well as old age itself at 1.04 times and Charlson at 1.14 times^31^. 

The variable number of adverse events was shown as a protection fator, reducing the risk of death by up to 88% in the occurrence of three or more no-ninfectious AE. It is believed that this peculiarity is due to the fact that the team pays greater attention during the care given to these patients in order to avoid the occurrence of new events. However, it must be emphasized that, regardless of the number of adverse events, its occurrence is associated with an increase in deaths in patients admitted to the ICU^15^. 

The impact of the occurrence of adverse events in intensive care is very large. Although there are few studies on this subject in Brazil, the results are worrisome, especially when considering the issue of underreporting that continues to occur due to fear of punishment or medical and legal sanctions and lack of surveillance ^(^ ^32^.

Considering the retrospective design of this study, we are not allowed to evaluate other information necessary to better explain the work and, despite the large number of adverse events identified, it is possible that this number is even greater when considering underreporting. 

However, as strengths, it is due to its outline that this study contributes in a positive way to allow inferences of its results, in addition to its unprecedented nature, since any work on the subject in the Amazon region is unknown, which favors the knowledge of the reality on this subject in Brazil.

## Conclusion

Patients who had suffered an adverse event and who were more than 50 years of age, with infection/sepsis, greater severity, i.e. SAPS 3 > 30 and Charlson > 1, presented a higher risk of death; however, the greater number of AEs did not contribute to increase the risk of death among the evaluated patients. 

 The notification of adverse events in the intensive care unit is an important way to control the quality of care, because the identification of failures allows investing in preventive measures and, thus, avoiding damages to the patients. In order to reduce the occurrence of non-infectious AEs, it is necessary to invest in qualification and updating of the professionals involved in the assistance, enough human resources to meet the demand, physical structure and adequate technology. 

## References

[1] World Health Organization (2012). Patient safety research: introductory course - Session 1. What is
patient safety?.

[2] Canineu R, Guimarães PH, Lopes R, Vendrame LS, Fonseca MA, Lopes AC (2006). Iatrogenic in Intensive Care Medicine. Rev Bras Ter Intensiva.

[3] Sommella L, Waure C, Ferriero AM, Biasco A, Mainelli MT, Pinnarelli L (2014). The incidence of adverse events in a Italian acute care hospital:
findings of a two-stage method in a retrospective cohort
study. BMC Health Serv Res.

[4] Mendes W, Travassos C, Martins M, Marques PM (2008). Adjustment of adverse events assessment forms for use in
Brazilian hospitals.. Rev Bras Epidemiol.

[5] Novaretti MCZ, Santos EV, Quitério LM, Daud-Gallotti RM (2014). Nursing workload and occurrence of incidents and adverse events
in ICU patients. Rev Bras Enferm.

[6] Donchin Y, Gopher D, Olin M, Badihi Y, Biesky M, Sprung CL (2003). A look into the nature and causes of human errors in the
intensive care unit. Qual Saf Health Care.

[7] Vlayen A, Verelst S, Bekkering GE, Schrooten W, Hellings J, Claes N (2012). Incidence and preventability of adverse events requiring
intensive care admission: a systematic review. J Eval Clin Pract.

[8] James JT (2013). A new, evidence-based estimate of patient harms associated with
hospital care. J Patient Saf.

[9] Austin M, Derk J Lives Lost, Lives Saved: A Comparative Analysis of Avoidable Deaths at
Hospitals Graded by The Leapfrog Group 2016.

[10] Makary MA, Daniel M (2016). Medical error-the third leading cause of death in the
US. BMJ.

[11] Aranaz-Andrés JM, Aibar-Remón C, Limón-Ramírez R, Amarilla A, Restrepo FR, Urroz O (2011). Prevalence of adverse events in the hospitals of five Latin
American countries: results of the ‘Iberoamerican Study of Adverse Events’
(IBEAS). BMJ Qual Saf.

[12] W, Martins M, Rozenfeld S, Travassos C (2009). The assessment of adverse events in hospitals in
Brazil. Int J Qual Health Care.

[13] Martins M, Travassos C, Mendes W, Pavão AL (2011). Hospital deaths and adverse events in Brazil. BMC Health Serv Res.

[14] Mendes W, Pavão ALB, Martins M, Moura MLO, Travassos C (2013). The feature of preventable adverse events in hospitals in the
State of Rio de Janeiro, Brazil. Rev Assoc Med Bras.

[15] Roque KE, Tonini T, Melo ECP (2016). Adverse events in the intensive care unit: impact on mortality
and length of stay in a prospective study. Cad Saúde Pública.

[16] Assad EC (2011). Erros e eventos adversos não infecciosos relacionados à assistência em
terapia intensiva de adultos [dissertação].

[17] Thomas EJ, Petersen LA (2003). Measuring errors and adverse events in health
care. J Gen Intern Med.

[18] Silva JM, Malbouisson LMS, Nuevo HL, Barbosa LGT, Marubayashi LY, Teixeira IC (2010). Applicability of the Simplified Acute Physiology Score (SAPS 3)
in Brazilian Hospitals. Rev Bras Anestesiol.

[19] Rothschild JM, Landrigan CP, Cronin JW, Kaushal R, Lockley SW, Burdick E (2005). The critical care safety study: the incidence and nature of
adverse events and serious medical errors in intensive care. Crit Care Med.

[20] Forster AJ, Kyeremanteng K, Hooper J, Kg Shojania, Van Walraven C (2008). The impact of adverse events in the intensive care unit on
hospital mortality and length of stay. BMC Health Serv Res.

[21] Depasse B, Pauwels D, Somers Y, Vincent JL (1998). A profile of European ICU nursing. Intensive Care Med.

[22] Conselho Federal de Enfermagem (2017). Resolução COFEN nº 543 18 de abril de 2017 que atualiza e estabelece
parâmetros para o Dimensionamento do Quadro de Profissionais de Enfermagem
nos serviços/locais em que são realizadas atividades de enfermagem.

[23] Agência Nacional de Vigilância Sanitária (2012). Resolução da Diretoria Colegiada. RDC nº 26 de 11 de maio de 2012.
Altera a Resolução RDC 7 de 24 de fevereiro de 2010 que dispõe sobre os
requisitos mínimos para funcionamento de unidades de terapia intensiva e da
outras providências.

[24] Garrouste-Orgeas M, Timsit JF, Vesin A, Schwebel C, Arnodo P, Lefrant JY (2010). Selected medical errors in the intensive care unit: results of
the IATROREF study: parts I and II. Am J Respir Crit Care Med.

[25] Ohta Y, Sakuma M, Koike K, Bates DW, Morimoto T (2014). Influence of adverse drug events on morbidity and mortality in
intensive care units: the JADE study. Int J Qual Health Care.

[26] Bezerra SMG, Pereira LC, Luz MHBA, Santana WS (2014). Incidence of pressure ulcers in an intensive care unit of a
public hospital. Rev Enferm UFPI.

[27] Pestana MP, dos Santos Vieira R (2012). Nursing actions in the prevention of pressure ulcers in
ICU. Rev Cient Enferm.

[28] Vilela RPB, Jericó MC (2016). Medication errors: management of the medication error indicator
toward a more safety nursing practice. Rev Enferm UFPE.

[29] Ministério da Saúde (2013). Agência Nacional de Vigilância Sanitária. Protocolo de segurança na
prescrição, uso e administração de medicamentos.

[30] Jaul E, Menczel J (2015). A comparative, descriptive study of systemic factors and survival
in elderly patients with sacral pressure ulcers. Ostomy Wound Manage.

[31] Rowe T, Araujo KLB, Van Ness PH, Pisani MA, Juthani-Mehta M (2016). Outcomes of Older Adults With Sepsis at Admission to an Intensive
Care Unit. Open Forum Infect Dis.

[32] Beccaria LM, Pereira RAM, Contrin LM, Lobo SMA, Trajano DHL (2009). Nursing care adverse events at an intensive care
unit. Rev Bras Ter Intensiva.

